# Effectiveness of safety-netting approaches for acutely ill children: a network meta-analysis

**DOI:** 10.3399/BJGP.2024.0141

**Published:** 2024-12-24

**Authors:** Ruben Burvenich, David AG Bos, Lien Lowie, Kiyano Peeters, Jaan Toelen, Laure Wynants, Jan Y Verbakel

**Affiliations:** Leuven Unit for Health Technology Assessment Research (LUHTAR), Department of Public Health and Primary Care, KU Leuven; Academic Centre for General Practice, Department of Public Health and Primary Care, KU Leuven, Leuven, Belgium.; Leuven Unit for Health Technology Assessment Research (LUHTAR), Department of Public Health and Primary Care, KU Leuven; Academic Centre for General Practice, Department of Public Health and Primary Care, KU Leuven, Leuven, Belgium.; Faculty of Medicine, KU Leuven, Leuven, Belgium.; Ordina, Sopra Steria, Diegem, Belgium.; Woman and Child, Department of Development and Regeneration, KU Leuven, Leuven, Belgium; Department of Pediatrics, University Hospitals Leuven, Leuven, Belgium.; LUHTAR, KU Leuven, Leuven, Belgium; Department of Epidemiology, Care and Public Health Research Institute, Maastricht University, Maastricht, The Netherlands.; Leuven Unit for Health Technology Assessment Research (LUHTAR), Department of Public Health and Primary Care, KU Leuven; Academic Centre for General Practice, Department of Public Health and Primary Care, KU Leuven, Leuven, Belgium.

**Keywords:** antibiotic resistance, child, communicable diseases, general practice, network meta-analysis, primary health care

## Abstract

**Background:**

Safety-netting advice (SNA) can help in the management of acutely ill children.

**Aim:**

To assess the effectiveness of different SNA methods on antibiotic prescription and consumption in acutely ill children.

**Design and setting:**

Systematic review and network meta-analysis of randomised controlled trials, cluster randomised trials, non-randomised studies of interventions, and controlled before–after studies in ambulatory care in high-income countries.

**Method:**

MEDLINE, Embase, Web of Science Core Collection, and Cochrane Central Register of Controlled Trials were searched (22 January 2024). Risk of bias (RoB) was assessed with Cochrane’s RoB 2 tool, the Revised Cochrane Tool for Cluster-Randomised Trials, and the Risk Of Bias In Non-randomised Studies — of Interventions tool. Certainty of evidence was assessed using the Confidence in Network Meta-Analysis approach. Sensitivity analyses and network meta-regression were performed.

**Results:**

In total, 30 studies (20 interventions) were included. Compared with usual care, paper SNA may reduce: antibiotic prescribing (odds ratio [OR] 0.66, 95% confidence interval [CI] = 0.53 to 0.82, *I*^2^ = 92%, very low certainty, three studies, 35 988 participants), especially when combined with oral SNA (OR 0.40, 95% CI = 0.08 to 2.00, *P*-score = 0.86); antibiotic consumption (OR 0.39, 95% CI = 0.27 to 0.58, low RoB, one study, 509 participants); and return visits (OR 0.74, 95% CI = 0.63 to 0.87). Compared with usual care, video SNA, read-only websites, oral SNA, and web-based SNA (in descending order of effectiveness) may increase parental knowledge (ORs 2.33–4.52), while paper SNA may not (ORs 1.18–1.62). Similarly, compared with usual care, video SNA and web-based modules may improve parental satisfaction (ORs 1.94–4.08), while paper SNA may not (OR 1.85, 95% CI = 0.48 to 7.08).

**Conclusion:**

Paper SNA (with oral SNA) may reduce antibiotic use and return visits. Video, oral, and online SNA may improve parental knowledge, whereas video SNA and web-based modules may increase parental satisfaction.

## Introduction

Most childhood infections are self-limiting, yet inappropriate antibiotic prescribing for acutely ill children remains high.^1^^,^^2^ This is associated with more return visits and antimicrobial resistance (AMR).^3^^,^^4^ Safety-netting advice (SNA) — informing carers of acutely ill children on the expected disease course and where, when, and/or how to find help^5^ — is a cheap and practical intervention that can help, especially when serious infections are not suspected and no antibiotics are required.^6^^,^^7^

The most effective components and ways of delivering SNA are currently unknown.^8^^,^^9^ Previous reviews are either outdated or do not focus on children,^6^^,^^10^^–^^15^ so research is needed to compare the effectiveness of different SNA strategies.^6^^,^^11^^,^^12^^,^^15^^,^^16^ As such, this study aims to investigate the impact of different SNA approaches and usual care on antibiotic prescribing/consumption, return visits, parental knowledge, and parental satisfaction for acutely ill children (aged ≤18 years) in ambulatory care in high-income countries.

## Method

This systematic review and network meta-analysis (NMA) is reported according to the PRISMA-NMA reporting guideline.^17^ The protocol was prospectively published on PROSPERO (reference: CRD42022356961).

### Search strategy, eligibility criteria, and study selection

A search was conducted (22 January 2024) of MEDLINE (PubMed), Embase, Web of Science Core Collection, and the Cochrane Central Register of Controlled Trials since inception using the terms ‘safety netting advice’, ‘acute illness’, ‘children 0–18 years’, and ‘ambulatory care’ and a specificity filter (see Supplementary Box S1). A manual reference search of articles included in this and similar review(s)^6^^,^^10^^–^^15^ was performed.

Included were randomised controlled trials (RCTs), cluster randomised trials (CRTs), non-randomised studies of interventions (NRSIs), or controlled before–after studies (CBAs) of acutely ill children aged ≤18 years presenting to ambulatory care in high-income countries^18^ that compared SNA interventions with usual care and/or each other. These study designs were chosen because individually RCTs have high internal validity and provide strong evidence for causal inference. In contrast, CRTs are more pragmatic, reflecting a more natural clinical setting compared to individually randomised trials. NRSIs and CBAs can provide additional evidence when RCTs are unavailable; however, this comes at the cost of a higher risk of bias (RoB), greater heterogeneity, and less robust evidence. The inclusion criteria for SNA interventions were based on mixed-methods research with experts and parents.^5^ These included:

- explaining the expected disease course;
- communicating diagnostic uncertainty;
- communicating when, where, and/or how to find help;
- delayed antibiotic prescribing strategies; and/or
- educational information on antibiotic misuse and AMR.

**Table:** How this fits in

Safety-netting advice (SNA) can help in the management of acutely ill children, but the best delivery methods were previously unclear. This research shows that paper SNA may reduce antibiotic prescribing, consumption, and return visits. Additionally, video SNA and web-based modules may improve parental knowledge and satisfaction. These findings highlight the practical benefits of SNA in optimising antibiotic use and improving outcomes for both sick children and their parents.

Outcomes of interest were antibiotic prescribing, antibiotic consumption, return visits, parental knowledge (after intervention or change from baseline), and parental satisfaction. Data on other outcomes, such as acute otitis media pain score, hospital admission rate, and parental anxiety, were also extracted. Language restrictions were not applied and DeepL Translate or Google Translate were used for translation. Studies targeting children with comorbidities or chronic diseases and interventions for healthcare professionals were excluded.

After removing duplicate records, four authors used Rayyan software (https://www.rayyan.ai) to independently screen all studies in pairs, by title and abstract, and by full text.

Disagreements were resolved through discussion or by another author.

### Data extraction

Data were extracted by two authors and checked by a third. Disagreements were resolved through discussion or by a fourth author. If needed, study authors were contacted for additional information or data. Outcome values were included if it was possible to calculate missing measures of variance based on sample sizes, 95% confidence intervals (CIs), ranges, or interquartile ranges (IQRs), and they were screened for data skewness.^19^^,^^20^ Missing means were substituted by medians if the distribution of the data was not skewed, otherwise they were excluded.^21^ If numeric outcome data were not available or were not received from study authors on request, these were estimated by two authors who visually inspected and directly measured figures and plots*.*

### RoB assessment

Two authors independently evaluated the within-study RoB for each outcome. Disagreements were resolved by another author through discussion. Cochrane’s RoB 2 was used for RCTs,^22^ the Revised Cochrane Tool for Cluster-Randomised Trials was used for CRTs,^23^ and the Risk Of Bias In Non-randomised Studies — of Interventions tool was used for NRSIs and CBAs.^24^

### Certainty of evidence

The Confidence in Network Meta-Analysis (CINeMA) approach^25^^–^^27^ and online platform (https://cinema.ispm.unibe.ch) were used to assess the certainty of evidence for all outcomes: within-study RoB, reporting bias, indirectness, imprecision (clinically important size defined as odds ratio [OR] >1.2 or OR <0.83), heterogeneity, and incoherence. With this software, related figures and plots were generated, and the per-study contribution to the pooled effect estimate was calculated. Using netsplit^28^ in R, the ratio of ratios and *P*-values of tests for disagreement between direct and indirect effects were calculated.

### Data analysis

If the transitivity assumption was met (by assessing study characteristics), frequentist NMA was performed; otherwise, studies were narratively reviewed. Transitivity refers to the assumption *‘that the different sets of randomized trials are similar, on average, in all important factors other than the intervention comparison being made’*, to justify making indirect comparisons.^29^ NMA is *‘a technique for comparing three or more interventions simultaneously in a single analysis by combining both direct and indirect evidence across a network of studies.’*^29^

*P*-scores were calculated to rank the effectiveness of interventions.^30^ For dichotomous outcomes, random-effects meta-analysis models on natural log ORs were used to calculate pooled effects with 95% CIs. Ordinal and measurement scale outcomes were dichotomised to use the data for NMA (cut-offs: ≥3 for a five-point Likert scale and ≥4 for a six-point Likert scale).^31^ In studies with continuous outcomes on different scales, the standardised mean difference (SMD)^19^^,^^31^ was calculated, and the transformed SMD was used as an estimate of the natural log OR assuming either normal or logistic (if skewed) distributions.^32^^,^^33^ To avoid cells with a value of 0 when calculating ORs, the modified Haldane-Anscombe correction was applied by adding 0.5 to all cells in the contingency table.^34^ Network plots were constructed and NMA geometry was reported as per Tonin *et al*’s work^35^ (see Supplementary Box S2).

Bayesian network meta-regression (NMR) was performed to assess the impact of study design, setting, RoB, and single versus compound intervention on effect sizes and network inconsistency.^36^ If there was a specific hypothesis on how publication bias might affect a network model with at least 10 studies, comparison-adjusted funnel plots were constructed and Egger’s regression test calculated.^37^^,^^38^ Sensitivity analyses were performed to assess the robustness of the results by excluding studies with high RoB and *n* = <500.

All analyses and figures not related to certainty of evidence were performed and generated with the netmeta (frequentist) package in R. Additional Bayesian NMA was performed using the GeMTC^39^ package and compared the direction and magnitude of the results with the frequentist NMA findings. Meta-regression is not yet possible in the frequentists netmeta package and, therefore, was performed in the Bayesian GeMTC package. Default vague Bayesian priors (determined from the data) were used.

## Results

**Figure 1.**
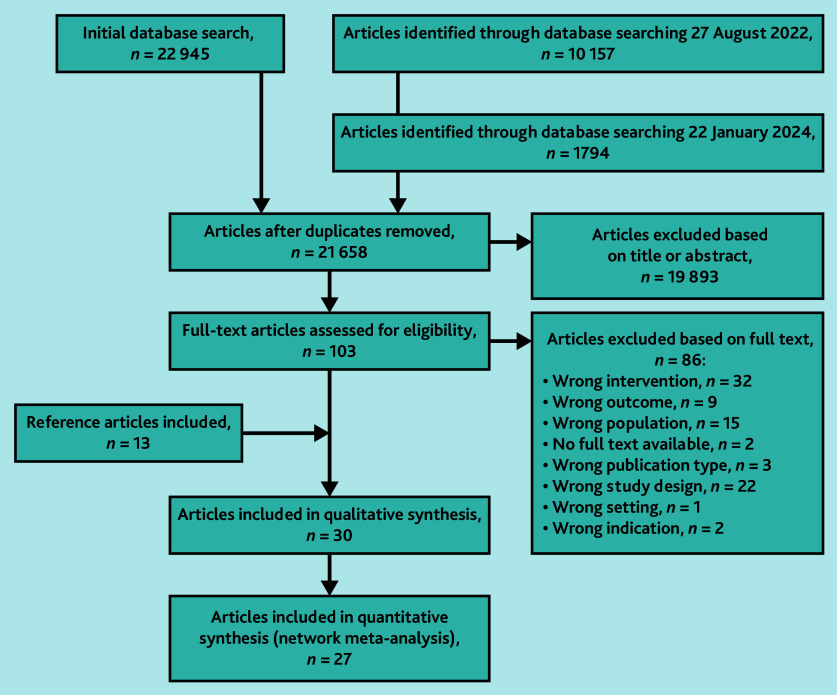
PRISMA flow chart of included studies. PRISMA = Preferred Reporting Items for Systematic reviews and Meta-Analysis.

An initial database search performed on 8 January 2021 yielded 22 945 articles. Updates on 27 August 2022 and 22 January 2024 added 10 157 and 1794 records, respectively. After removing duplicates, a total of 21 658 records remained. From these, 30 studies were included in the qualitative synthesis. Of the 30 studies, 27 were included in the NMA, as the standard error could not be calculated for three studies (Figure 1).

### Characteristics of included studies

In total, 22 RCTs,^40^^–^^61^ five CRTs,^62^^–^^66^ and three NRSIs^67^^–^^69^ from 11 different countries (14/30 from the ^US40,^^41^^,^^43^^–^^47^^,^^51^^,^^57^^,^^58^^,^^61^^,^^67^^–^^69^) were included (see Supplementary Table S1). Seventeen studies were conducted in a paediatric emergency department^40^^,^^41^^,^^43^^,^^44^^,^^46^^–^^49^^,^^51^^–^^54^^,^^59^^,^^61^^,^^67^^–^^69^ and 13 in a general practice, paediatric, primary care, or combined setting.^42^^,^^45^^,^^50^^,^^55^^–^^58^^,^^60^^,^^62^^–^^66^ Respiratory tract infections (RTIs) were the most common condition.

### RoB and certainty of evidence

The overall RoB of the 81 assessed outcomes (22 RCTs) was ‘low’ for 19 outcomes, ‘high’ for 13, and there were ‘some concerns’ for 49 (see Supplementary Tables S2 and S3). High RoB in five studies^40^^–^^44^ was often due to issues with the randomisation process. For the 26 outcomes of the five CRTs, RoB was ‘low’ for 14 outcomes, ‘high’ for one, and there were ‘some concerns’ for 11. All seven outcomes of the three NRSIs had a ‘serious’ RoB, mainly due to missing data bias.

Overall, evidence certainty ranged from moderate to very low, primarily due to incoherence and heterogeneity concerns. Supplementary Table S4 features detailed results, ratio of ratios, and *P*-values for disagreement tests between direct and indirect effects.

### Primary outcomes

#### Antibiotic prescribing

**Figure 2.**
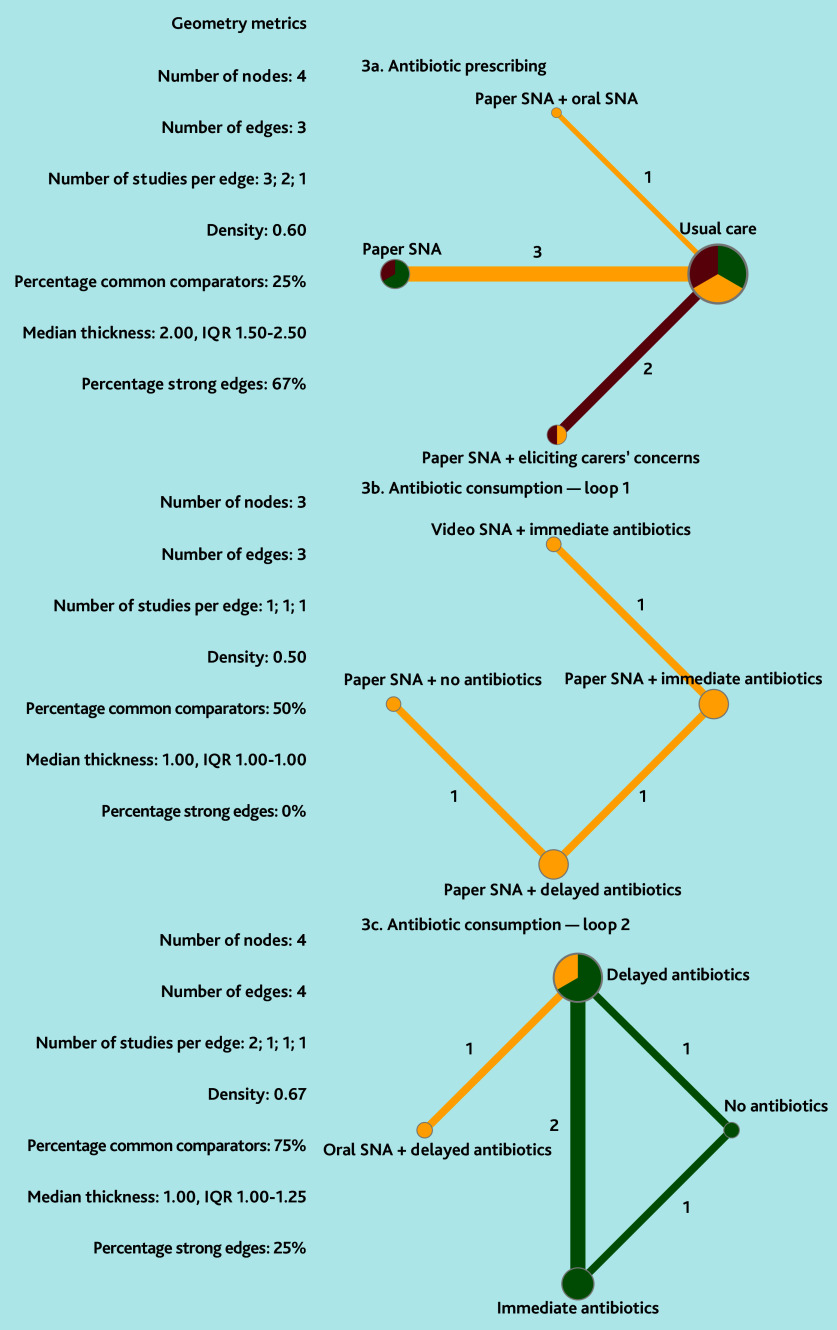
Network plots (with geometry metrics) of direct evidence of the primary outcomes. Node (circle) size and edge (connecting line) width correspond to the number of studies per intervention or comparator and the effect assessment between them, respectively. Likewise, the color of the nodes represents the risk of bias of the contributing studies, while the edge color reflects the average risk of bias across studies comparing two interventions. IQR = interquartile range.

The certainty of evidence for all primary outcomes was low to very low (Table 1 and Figure 2). Paper SNA was associated with a reduction in antibiotic prescribing compared with usual care (OR 0.66, 95% CI = 0.53 to 0.82) and paper SNA plus eliciting carers’ concern (OR 0.35, 95% CI = 0.23 to 0.53). Paper SNA plus eliciting carers’ concern was associated with higher antibiotic prescribing than usual care (OR 1.89, 95% CI = 1.34 to 2.67). Paper SNA plus oral SNA may be associated with a difference in antibiotic prescribing compared with usual care (OR 0.40, 95% CI = 0.08 to 2.00), but not compared with other SNA methods (ORs 0.66–4.76), although it was ranked most effective in the network (*P*-score = 0.86). All forest plots and netsplit plots are shown in Supplementary Figure S1. The study by de Bont *et al*^63^ demonstrated that, compared with usual care, the actual use of paper SNA (OR 0.83, 95% CI = 0.74 to 0.94) significantly reduced antibiotic prescribing, whereas simply having access to paper SNA (OR 0.90, 95% CI = 0.79 to 1.02) did not show a statistically significant effect.

**Table 1.** Findings on interventions and antibiotic prescribing

	**Odds ratio (95% CI), certainty of evidence, heterogeneity (if appliable), direct versus indirect versus mixed evidence, number of studies, number of participants**		

	**Paper SNA**	**Paper SNA + ECC**	**Paper + oral SNA**
**Paper SNA + ECC**	0.35 (0.23 to 0.53) 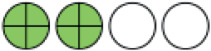 , only indirect evidence, five studies,^42^^,^^62^^–^^65^ 37 504 participants		
**Paper + oral SNA**	1.67 (0.33 to 8.47) 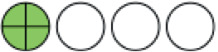 , only indirect evidence, four studies,^42^^,^^45^^,^^63^^,^^64^ 19 708 participants	4.76 (0.92 to 24) 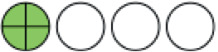 , only indirect evidence, three studies,^45^^,^^62^^,^^65^ 1552 participants	
**Usual care**	0.66 (0.53 to 0.82) 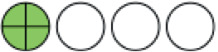 , *I*^2^ = 92%, only direct evidence, three studies,^42^^,^^63^^,^^64^ 35 988 participants	1.89 (1.34 to 2.67) 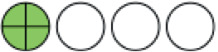 , *I*^2^ = 0%, only direct evidence, two studies,^62^^,^^65^ 1516 participants	0.40 (0.08 to 2.00) 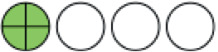 , only direct evidence, one study, ^45^ 36 participants

*ECC = eliciting carers’ concern.* 
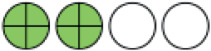
 *= low certainty evidence. SNA = safety-netting advice.* 
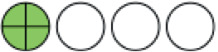
 *= very low certainty evidence.*

#### Antibiotic consumption

The available evidence allowed two network loops to be constructed with one separate study. Due to data sparsity, evaluating the certainty of evidence for loop 1 was not deemed useful.

In loop 1, paper SNA plus delayed antibiotics and paper SNA plus no antibiotics both reduced antibiotic consumption compared with paper SNA plus immediate antibiotics (OR 0.01, 95% CI = <0.01 to 0.02 and OR 766, 95% CI = 158 to 3706, respectively), and compared with video SNA plus immediate antibiotics (OR 0.002, 95% CI = <0.001 to 0.02 and OR 0.001, 95% CI = <0.001 to 0.004, respectively) (some RoB) (Table 2). There was no statistically significant difference between the outcomes of paper SNA plus immediate antibiotics and video SNA plus immediate antibiotics (OR 0.49, 95% CI = 0.16 to 1.53). A statistically significant difference was observed between paper SNA plus no antibiotics and paper SNA plus delayed antibiotics (OR 3.67, 95% CI = 1.95 to 6.87) (some RoB).

**Table 2.** Findings on interventions and antibiotic consumption: loops 1 and 2

	**Odds ratio (95% CI), certainty of evidence, heterogeneity (if appliable), direct versus indirect versus mixed evidence, number of studies, number of participants**		

**Loop 1**			

	**Paper SNA + delayed AB**	**Paper SNA + immediate AB**	**Paper SNA + no AB**
**Paper SNA + immediate AB**	0.01 (<0.01 to 0.02) 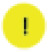 , only direct evidence, one study,^55^ 284 participants		
**Paper SNA + no AB**	3.67 (1.95 to 6.87) 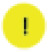 , only direct evidence, one study^51^ 206 participants	766 (158 to 3706) 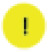 , only indirect evidence, two studies,^51^^,^^55^ 490 participants	
**Video SNA + immediate AB**	0.002 (<0.001 to 0.02) 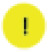 , only indirect evidence, two studies,^49^^,^^55^ 433 participants	0.49 (0.16 to 1.53) 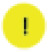 , only direct evidence, one study,^49^ 149 participants	0.001 (<0.001 to 0.004) 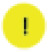 , only indirect evidence, three studies,^49^^,^^51^^,^^55^ 639 participants

**Loop 2**			

	**Delayed AB**	**Immediate AB**	**No AB**

**Immediate AB**	27 (4.52 to 162) 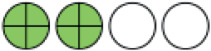 , *I*^2^ = 91%, only direct evidence, two studies,^50^^,^^61^ 559 participants		
**No AB**	0.26 (0.03 to 2.71) 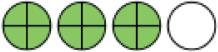 , mixed evidence, two studies,^50^^,^^61^ 559 participants	0.01 (<0.01 to 0.11) 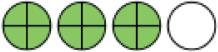 , mixed evidence, two studies,^50^^,^^61^ 559 participants	
**Oral SNA + delayed AB**	0.11 (0.01 to 1.55) 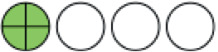 , only direct evidence, one study,^60^ 81 participants	0.004 (<0.001 to 0.10) 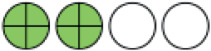 , only indirect evidence, three studies,^50^^,^^60^^,^^61^ 640 participants	0.42 (0.01 to 14) 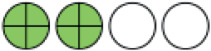 , only indirect evidence, three studies,^50^^,^^60^^,^^61^ 640 participants

AB = antibiotics. 
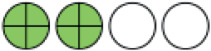
 = low certainty evidence. 
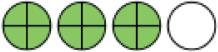
 = moderate certainty evidence. SNA = safety-netting advice. 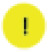 = some risk of bias. 
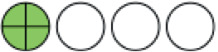
 = very low certainty evidence.

**Figure 3.**
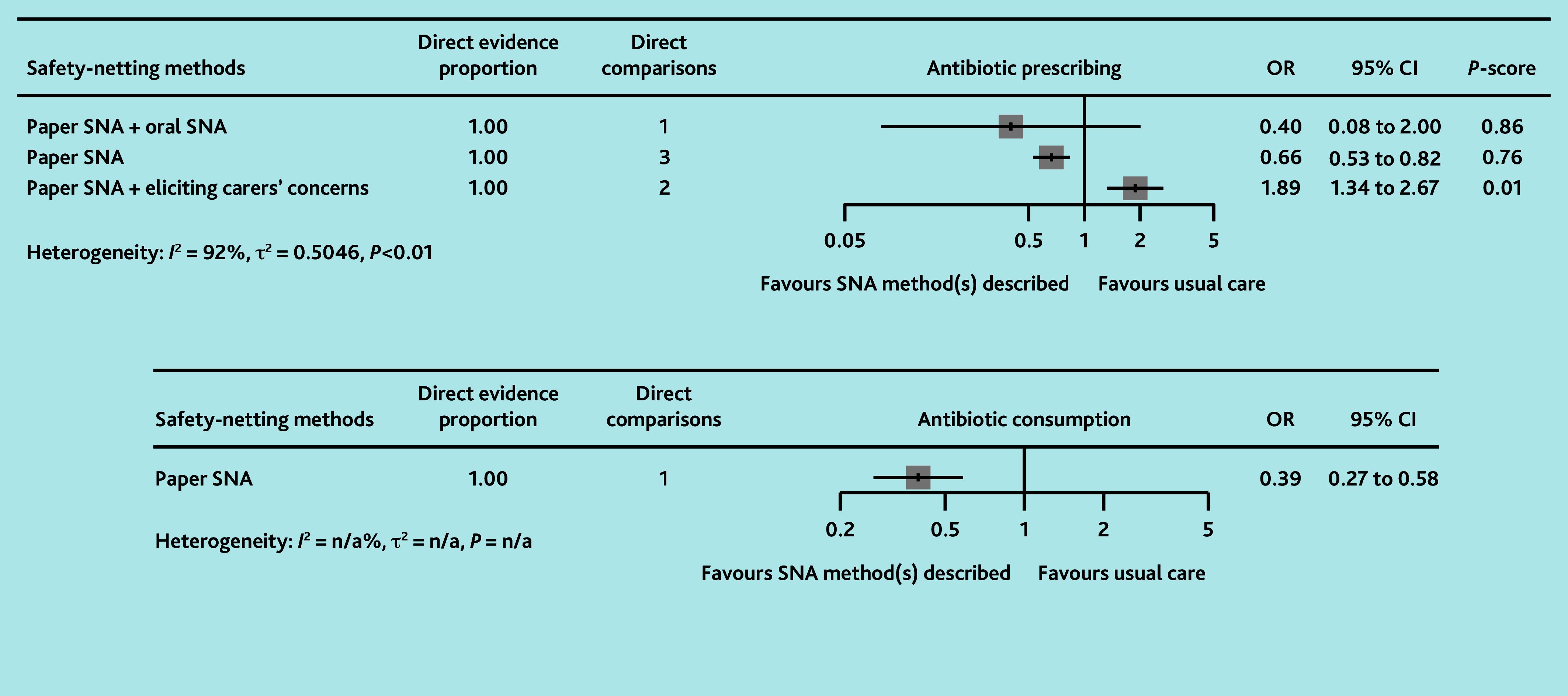
Forest plots of network analyses for the primary outcomes with usual care as the comparator. OR = odds ratio. SNA = safety-netting advice.

In loop 2, compared with immediate antibiotics, oral SNA plus delayed antibiotics (OR 0.004, 95% CI = <0.001 to 0.10), delayed antibiotics (OR 27, 95% CI = 4.52 to 162), and no antibiotics (OR 0.01, 95% CI = <0.01 to 0.11) were all associated with lower antibiotic consumption (low to moderate certainty evidence) (Table 2). There were no statistically significant differences between oral SNA plus delayed antibiotics and delayed antibiotics alone (OR 0.11, 95% CI = 0.01 to 1.55) or no antibiotics (OR 0.42, 95% CI = 0.01 to 14), with the certainty of evidence ranging from very low to low. The study by Francis *et al*^64^ found that paper SNA was likely associated with significantly less antibiotic consumption than usual care (OR 0.39, 95% CI = 0.27 to 0.58, low RoB) (Figure 3).

### Secondary and other outcomes

Among SNA methods, paper SNA was the only one shown to reduce return visits compared with usual care (OR 0.74, 95% CI = 0.63 to 0.87) (see Supplementary Figure S2). Return visit results from Baker *et al*^47^ (paper SNA versus usual care) and Hartling *et al*^48^ (video SNA versus usual care) were excluded from the pooled analyses due to their substantially longer follow-up times (319–656 days and 1 year, respectively), violating the transitivity assumption. Both studies found no differences between intervention and control groups at these extended follow-up times.

Compared with usual care, video SNA, read-only websites, oral SNA, and web-based SNA (in descending order of effectiveness) may increase parental knowledge (ORs 2.33–4.52), while paper SNA may not (ORs 1.18–1.62). Similarly, compared with usual care, video SNA and web-based modules may improve parental satisfaction (ORs 1.94–4.08), while paper SNA may not (OR 1.85, 95% CI = 0.48 to 7.08) (see Supplementary Figure S2). A descriptive synthesis for other outcomes — such as acute otitis media pain score, hospital admission rate, and parental anxiety — is given in Supplementary Box S3.

### Additional analyses

Funnel plots were not constructed as the criteria were not met. Bayesian NMA results confirmed the direction and magnitude of the frequentist NMA findings, with 95% credible intervals (CrIs) being generally wider than the corresponding 95% CIs (see Supplementary Table S5). None of the NMR models were statistically significant for any covariate (see Supplementary Box S4). Sensitivity analyses showed no different results (see Supplementary Figure S3).

## Discussion

### Summary

Compared with usual care: paper SNA may reduce antibiotic prescribing (especially when combined with oral SNA), antibiotic consumption, and return visits; video SNA, read-only websites, oral SNA, and web-based SNA may increase parental knowledge; and video SNA (with conflicting results) and web-based modules may improve parental satisfaction.

NMR showed that the observed heterogeneity cannot be explained by the covariates analysed, although each NMR contained ≤10 studies, which may be insufficient data to detect intervention by covariate interactions. Bayesian NMA results confirmed the findings; the 95% CrIs were wider than the 95% CIs, but this is to be expected, especially when non-informative priors are used. Sensitivity analyses indicated that the findings of the main analyses were robust.

### Strengths and limitations

A highly sensitive database search, including records with all types of SNA interventions and outcomes was performed. The diversity of interventions was used to the authors’ advantage by performing NMA, carefully assessing which studies to include in the networks (transitivity assumption), thereby increasing the reliability of the results. Sensitivity analyses and NMR were also performed, using dedicated RoB tools to assess the certainty of evidence.

Potential limitations include not searching grey-literature databases, which may introduce publication bias; however, the main interest was in peer-reviewed publications. Due to between-intervention heterogeneity (intransitivity), the authors could only construct several smaller networks for each outcome, thereby limiting comparability and reducing the certainty of evidence. Incoherence in the resulting networks was often noted. There was substantial within-intervention heterogeneity; for example, the paper SNA category includes different pamphlets or written SNA resources. Intransitivity also arose from using different scales for parental knowledge and satisfaction, some of which were dichotomised, potentially acting as effect modifiers. Performance bias in individual studies was unavoidable due to the nature of the interventions.

### Comparison with existing literature

The results presented here align with those of de Bont *et al,*^13^ who evaluated the effectiveness of paper SNA for common infections (*n* = 8, GP setting, all ages). Similar to the findings presented here, Andrews *et al*^15^ showed that paper SNA can reduce consultations (all ages). They stated that delayed prescribing could halve antibiotic use;^15^ the results presented here differ, because more recent studies^49^^,^^50^ were included and because Andrews *et al*^15^ pooled the results of studies with considerably different interventions and controls (clinical heterogeneity). This illustrates the present NMA’s ability to combine direct and indirect evidence to simultaneously compare multiple interventions, a feature Chaimani *et al*^29^ highlight as a key strength of NMAs.

McDonagh *et al*^12^ assessed clinic-based patient education intervention studies (*n* = 5, RTIs, all ages) and found reductions in antibiotic prescribing and consumption; their findings on parental knowledge, parental satisfaction, and return visits align with those presented here. A Cochrane systematic review^6^ (*n* = 2, upper RTIs, children) found paper SNA may reduce antibiotic prescribing and consumption compared with usual care, but resulted in no changes in return visits and patient satisfaction. Mortazhejri *et al*^14^ found that patient-oriented interventions, especially delayed prescriptions, may reduce antibiotic use (upper RTIs, all ages). As with Andrews *et al*’s study,^15^ different interpretations and classifications of heterogeneous interventions and comparisons were observed in the study presented here.

### Implications for research and practice

The use of paper SNA is to be encouraged, as it can reduce antibiotic prescribing, consumption, and return visits, although it did not improve parental knowledge or satisfaction. Video SNA and online materials did succeed in this regard, but their effect on antibiotic prescribing is less known. A combination of paper and oral SNA may be the most effective approach to reduce antibiotic prescribing.

Moving forward, high-quality CRTs/RCTs with long follow-up periods are needed to assess different SNA approaches (coproduced by parents and experts) on multiple outcomes (patient oriented, provider oriented, and adverse events). Further study is also required on the cost-effectiveness of interventions, effects on antibiotic resistance of regional pathogens, and the impact of withdrawing the intervention.

## Data

Most of the relevant data are included within the article or as supplementary data. Additional data are available on reasonable request from the corresponding author.
